# Hereditary alpha tryptasemia and food allergy

**DOI:** 10.3389/falgy.2025.1583462

**Published:** 2025-05-20

**Authors:** Abigail Lang

**Affiliations:** Division of Allergy and Immunology, Department of Pediatrics, Ann and Robert H. Lurie Children's Hospital of Chicago, Chicago, IL, United States

**Keywords:** food allergy, hereditary alpha tryptasaemia, *TPSAB1* gene, food allergy severity, biomarker, alpha-tryptasemia

## Abstract

Food allergy (FA) and hereditary alpha-tryptasemia (HαT) are both relatively common conditions, but potential associations between these diagnoses have not been well-studied. Prior studies have suggested that acute rises in tryptase following food allergy reactions may not be as significant as reactions triggered by venom or drug allergy, but preliminary evidence suggests that the presence of α-tryptase and HαT is a risk factor for more severe reactions to foods. This mini review summarizes the epidemiology and diagnostic considerations of FA for patients with co-morbid HαT, potential effect of α-tryptase on food allergy reaction severity, and implications of tryptase genotyping in the management of FA. Additional research is needed to further investigate the relationship between FA and HαT.

## Introduction

Food allergy (FA) affects a significant proportion of the population, and allergic reactions to foods are one of the leading causes of anaphylaxis (1, 2). FA diagnoses are associated with decreased quality of life (3, 4), and there are no reliable or validated biomarkers to predict food allergy reaction severity. As the prevalence of food allergy continues to increase (5, 6), many patients with adverse reactions to food will also likely be found to have hereditary alpha-tryptasemia (HαT) given its relative frequency in the general population (7, 8). HαT is a genetic trait due to the presence of extra copies of the α-tryptase encoding gene *TPSAB1*. Patients with HαT have been shown to have higher rates of severe anaphylaxis (9), suggesting that patients with both FA and HαT may also be at increased risk of more severe food allergy reactions. Additionally, patients with clonal mast cell disorders such as systemic mastocytosis (SM) have higher rates of co-morbid HαT as compared to the general population, and hypersensitivity reactions to venom, drugs, and food triggers are frequently reported in this population. Patients with SM and HαT also have more severe anaphylaxis symptoms as compared to patients with SM or HαT alone, suggesting that *TPSAB1* copy number variants encoding α-tryptase contribute to allergic reaction severity (9, 10). A recent study also suggests that patients with history of anaphylaxis to medications, particularly antibiotics and monoclonal antibodies (mAbs), have higher rates of HαT than the general population (11). Given these findings, HαT is a promising biomarker for anaphylaxis severity and may play a role in the pathogenesis of FA hypersensitivity reactions. However, there remains a paucity of literature specifically investigating the associations between FA and HαT (Table 1).

**Table 1:** Summary of published literature on the association between food allergy and hereditary alpha-tryptasemia.

Authors	Country	Year	Key findings related to food allergy	Limitations
Lyons et al	Italy, Slovenia, United States	2021	Of 10 patients with both SM and HαT, nine had a history of systemic anaphylaxis with one of the subjects reporting anaphylaxis triggered by food. The authors noted that no specific cause or trigger of anaphylaxis, including FA, was increased by the presence of HαT.	Limited discussion of FA reactions, as focus of paper was on association of HαT with increased severity of *Hymenoptera* anaphylaxis in addition to notably high prevalence rates of HαT in patients with systemic mastocytosis and idiopathic anaphylaxis.
Giannetti et al	United States	2021	Of 101 patients with HαT in a retrospective study at Brighan and Women's Hospital, 58 subjects had a history of physician-confirmed anaphylaxis. Food was the second most common trigger for anaphylaxis in this cohort (29%).	No data about severity of FA reactions or types of food triggers.
Glover et al	Multiple	2021	In a review of published studies on the clinical phenotype of HαT, the reported prevalence of GI food sensitivities varied from 0 to 39% depending on the study.	Limited information about types of GI symptoms and food triggers
Robey et al	United Kingdom	2020	In a British cohort of patients with HαT, 39% reported food intolerances.	No distinctions made between OFC confirmed IgE-mediated FA reactions or other types of food sensitivities
Lyons	Multiple	2018	Review of clinical manifestations of HαT with reported increased rates of EGID and food intolerances in highly symptomatic patients in families with increased *TPSAB1* copy number.	Limited case series/reports of families with HαT
Chantran et al	France	2023	Of three siblings with peanut allergy, two with HαT had lower threshold for reactivity to peanut as well as higher levels of specific IgE to whole peanut and Ara h 2 component.	Case report
Greiner et al	Austria	2021	In a subset of patients with mastocytosis and reported history of allergy, 6 of 28 patients without co-morbid HαT reported allergic reactions to food, while 1 of 11 patients with mastocytosis and HαT had FA.	No details about reaction types or severity of reactions for patients with FA; lower rates of FA seen in patients with HαT which may have been due to small sample size
Constantine et al	United States	2024	Increased basal serum trptase levels were seen in 16% (15/92) subjects with EGID but <1% (1/143) of patients with atopic dermatitis with eosinophilia. Of the 15 subjects with EGID, 4 patients were found to have HαT as cause of elevated basal tryptase.	Small sample size; no investigation of co-morbid IgE-mediated FA or specific food triggers for EGID and relationship to baseline tryptase levels
Lang et al	United States	2023	Presence of α-tryptase and HαT correlated with increased rates of anaphylaxis to food and increased severity of food-triggered reactions in group of children who reacted at peanut food challenge as well as in replication cohort from the NIAID	Relatively low proportion of patients with HαT in both cohorts; few severe FA reactions with lower respiratory or cardiovascular symptoms reported

SM, systemic mastocytosis; FA, food allergy; HαT, hereditary alpha-tryptasemia; GI, gastrointestinal; OFC, oral food challenge; EGID, eosinophilic gastrointestinal disorders; NIAID, National Institute of Allergy and Infectious Disease.

## Food allergy reactions in patients with hereditary alpha-tryptasemia

Anaphylaxis triggered by food has been reported in cohorts of patients with HαT (9, 12, 13). Additionally, many patients with HαT also report history of food sensitivities (7, 8, 10, 14). In a British cohort of patients with HαT, 39% reported history of food intolerance (7), although there were not distinctions made between oral food challenge (OFC) confirmed IgE-mediated food allergy reactions or other food-induced sensitivities. Preliminary studies have also shown that patients with eosinophilic gastrointestinal disorders (EGID) also have higher baseline serum tryptase levels as compared to atopic controls and the general population, which may be due at least in part to an increased percentage of individuals with concomitant HαT (15). Additional investigation of the prevalence of FA in patients with HαT needs to be assessed, but the prevalence of food-triggered reactions in patients with HαT seems to be at least the same or increased as compared to the general population.

## Role of tryptase in food allergy reactions

Tryptase is often elevated in the acute period after anaphylaxis triggered by drugs or venom stings, and it is well known that HαT is associated with an increased risk for systemic anaphylaxis, particularly secondary to *Hymenoptera* venom stings (9). Conversely, prior reports suggest that tryptase may not rise or be as significant during severe food allergy induced anaphylaxis (16). However, in a more recent smaller study of children with food allergy, higher baseline serum tryptase correlated with severity of food allergy reactions (17). Additionally, in a cohort of adult subjects undergoing shrimp OFC, the ratio of peak tryptase after reaction divided by the baseline tryptase level demonstrated good sensitivity and specificity for the confirmation of anaphylaxis (18). Similarly, in a prospective cohort of adult patients with peanut allergy who underwent OFC, a rise in tryptase level of 30% or more from baseline was seen in patients who reacted during challenge, and increased peak levels correlated with reaction severity (19). Notably, in all these small cohort studies, there were few patients with elevated baseline tryptase levels, and the presence of HαT was not assessed. Despite these limitations, these studies suggest that tryptase does rise during acute food allergy reactions, though to a lesser degree than with drug or venom reactions. It should be noted that obtaining tryptase levels immediately before and after FA reactions is not feasible in routine clinical practice. Tryptase genotyping as a biomarker or surrogate for the tryptase level may be an alternative strategy that can ameliorate the difficulty in obtaining tryptase levels during acute reactions and the inherent variations in rise of tryptase depending on the allergen trigger. Additional studies of patients with FA and elevated baseline tryptase levels secondary to HαT will help to elucidate the role tryptase plays in FA reactions.

## Association between severity of food allergy reactions and hereditary alpha-tryptasemia

Few published reports specifically investigate the relationship of tryptase genotypes, HαT, and FA. In a case report from France, two siblings with peanut allergy and HαT reacted at lower doses of peanut allergen and had higher specific IgE (sIgE) to both whole peanut and Ara h2 and h6 components as compared to a third sibling from the same family who also had peanut allergy but not HαT, suggesting that HαT and increased α-tryptase may contribute to a more severe FA phenotype (20). To date, there has only been one published study specifically investigating the relationship between FA and HαT (21). In this study, the authors investigated the tryptase genotypes of two cohorts of patients, including an observational group of patients with food allergy and a smaller subset of children with peanut allergy who had reacted to peanut OFC. There were no significant differences in results of food allergy testing (sIgE or skin prick wheal size) or presence of additional atopic conditions between patients with different tryptase genotypes. In the observational cohort, the presence of any α-tryptase was associated with increased risk of anaphylaxis to food as compared to those who only had β-tryptase. Similarly, in the cohort of patients with peanut allergy, subjects who had any α-tryptase isoforms were more likely to have severe reactions at OFC than subjects with only β-tryptase isoforms. Additionally, in the peanut allergy cohort, there was a positive correlation between number of α-tryptase copies and food allergy reaction severity scores, suggesting a gene dosage effect. Notably, of the total 119 patients between the two cohorts, there were only five subjects with HαT (4%), but four out of five of these patients had history of a severe food allergy reaction. Studies have demonstrated that αβ-tryptase heterotetramers, the formation of which relies on the relative copy number of α-tryptase and β-tryptase encoding genes and is increased in HαT, contribute to allergic reaction severity (9, 22) which provides a potential mechanism for the more severe reactions associated with increased relative α-tryptase copy number. Given this preliminary data, the presence of α-tryptase appears to confer a higher risk of severe FA reactions in a gene-dosage dependent manner. Interestingly, this tendency for subjects with increased α-tryptase copies to have more severe reactions was similarly seen in a cohort of patients with drug-induced anaphylaxis (11) further supporting the gene-dosage effect of α-tryptase on allergic reaction severity. Larger studies of patients with both FA and HαT will provide more clarity about the magnitude of this effect.

## Discussion

While the prevalence of FA in patients with HαT seems to be similar to the general population, there is preliminary evidence that the presence of α-tryptase and HαT is a risk factor for more severe reactions to foods, similar to the increased risk of severe reactions with *Hymenoptera* venom allergy and idiopathic anaphylaxis. Clinicians should consider tryptase genotyping as a biomarker for FA reaction severity, especially as risk stratification will likely become more important and essential to counseling patients and families as proactive FA management strategies such as immunotherapy modalities and biologic therapies for FA treatment become more universal (23, 24). While additional studies focused on the associations between FA and HαT are still needed, tryptase genotyping as a biomarker for food allergy reaction severity is promising.
